# Identification of AAAS gene mutation in Allgrove syndrome: A report of three cases

**DOI:** 10.3892/etm.2015.2677

**Published:** 2015-08-10

**Authors:** WENJING LI, CHUNXIU GONG, ZHAN QI, DI WU, BINGYAN CAO

**Affiliations:** Department of Endocrinology, Genetics and Metabolism, Beijing Children's Hospital, Capital Medical University, Beijing 100045, P.R. China

**Keywords:** Allgrove syndrome, AAAS gene, genetic analysis, Chinese

## Abstract

Allgrove syndrome (AS) is an autosomal recessive congenital disease, caused by mutations in the AAAS gene, and is characterized by the triad of Addison's disease, achalasia and alacrima. The present study describes three newly diagnosed cases of AS, in which genetic analysis of the AAAS gene was used to identify AAAS gene mutations, to enhance the understanding of the pathogenesis and clinical manifestations of AS in the Chinese population. Two of the cases exhibited homozygous mutations of c.771delG (p.Arg258GlyfsX33) in exon 8 and one case exhibited a homozygous mutation of c.1366C>T (p.Q456X) in exon 15. A review of the current literature suggests that the AAAS c.771delG mutation has only been reported in the Chinese population. Genetic analysis of the AAAS gene in Chinese AS patients at a young age may facilitate an earlier diagnosis and the timely initiation of the appropriate treatment, ultimately improving the patient outcome.

## Introduction

Allgrove syndrome (AS; OMIM no. 231550), first described in 1978 (1), is a rare autosomal recessive congenital disease characterized by the triad of adrenal insufficiency (Addison's disease), failure of the lower esophageal sphincter to relax (achalasia) and absence of tear secretion (alacrima). AS is also known as 3A syndrome or, with the addition of autonomic and central nervous system impairment, 4A syndrome (2). The syndrome usually presents itself in the first decade of an individual's life, but cases have been also identified in adulthood, usually due to childhood symptoms having been overlooked. AS occurs worldwide, and its various clinical manifestations range from mild to severe (occasionally fatal) (1–4). The 3A syndrome is a progressive disorder and it can take years for the full clinical effects to develop (5). The pathogenic gene of AS, AAAS, is located on chromosome 12q13, consists of 16 exons and encodes the protein ‘alacrima, achalasia, adrenal insufficiency and neurologic disorder’ (ALADIN). The gene has been found to be ubiquitously expressed throughout the body and not just in the adrenal glands, as previously believed (6). A variety of mutations scattered throughout the gene have been reported, except in exon 3 (7). There does not appear to be a correlation between the different mutations found within AAAS and the clinical manifestations of the disease (6). In 2006, the Beijing Children's Hospital of Capital Medical University (Beijing, China) reported the first case of AS in the Han population from the mainland of China (8). Two additional cases (one male and one female patient) were admitted in 2012. In the present study, the clinical characteristics of these 3 cases were summarized and the literature was reviewed in order to enhance the understanding of the pathogenic and clinical characteristics of AS in the Chinese population and to improve diagnosis of the disease.

## Case report

### 

#### Methods

The present study comprises a retrospective analysis of 3 cases of AS diagnosed in 2006 or 2012. Written informed consent for all tests and treatments, as well as the genetic analysis, was provided from the parents of the patients. Whole blood samples were obtained from the patients and their parents for the AAAS gene analysis. Table I provides a summary of the symptoms and results of the genetic analysis for each case. Genomic DNA was isolated from peripheral blood using the Takara Blood Genome DNA Extraction kit (Takara Bio, Inc., Otsu, Japan). The entire coding region of the AAAS gene (NM_015665) was amplified and sequenced using the Sanger method (9). The primers and sequencing conditions were as previously reported (10). The patients' sequences were blasted against the GenBank Database, and the mutations in the gene of each patient were confirmed by sequencing the same fragment of their parents' DNA (NM_015665).

#### Patient 1

A Chinese girl, aged 7 years and 3 months, was diagnosed with AS on February 11, 2006. This was the first case of AS to be diagnosed on the mainland of China, to the best of our knowledge (6). Two years and 9 months prior to diagnosis, the patient had been admitted to a local hospital due to generalized hyperpigmentation and emaciation, in addition to a 3-min period of unconsciousness. The laboratory examination revealed that the blood electrolyte and serum cortisol levels of the patient, as well as the liver and kidney function, were normal; however, the levels of adrenocorticotropin (ACTH) were markedly elevated. A computed tomography scan of both adrenal glands and a magnetic resonance imaging (MRI) scan of the head revealed no significant anomalies. The patient was diagnosed with Addison's disease, and the hyperpigmentation slowly dissipated with oral hydrocortisone acetate (50 mg/m^2^).

Nine months prior to admission to the Beijing Children's Hospital, the patient had a vomiting incident without abdominal pain or regurgitation, which lasted 5–6 days. Treatment with an increased dose of hydrocortisone was not effective, and the patient exhibited increasing levels of weakness, fatigue, difficulty in eating and weight loss.

**Figure 1.**
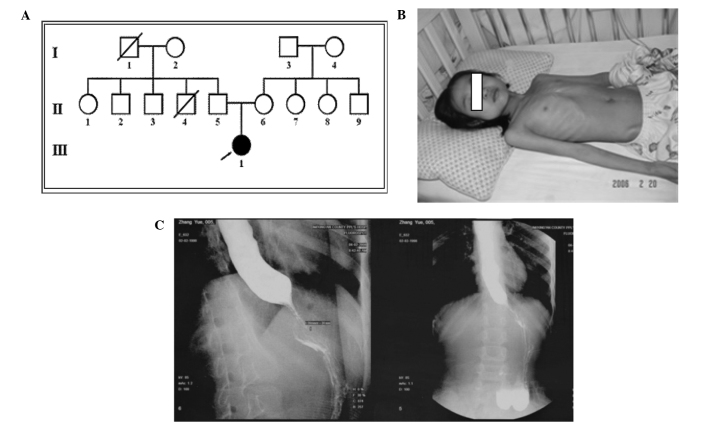
Patient 1. (A) Family pedigree for the 7-year-old female patient. The parents are the third generation of consanguineous marriages; they exhibit no clinical symptoms, and there are no other known AS patients in the family. (B) Admission of the patient to the hospital. Clinical manifestations included marked emaciation, a thin layer of subcutaneous fat, hyperpigmentation, and hand and upper limb muscle atrophy. (C) Gastrointestinal barium images of the patient. Upper gastrointestinal barium images revealed a characteristic ‘bird's beak’ appearance with esophagectasia and hyperanakinesis. The contrast agent was difficult to pass due to the delayed opening of the cardia.

The parents of the patient were healthy (father, 30 years old; mother, 32 years old). Gravida 1 (G1) and (G2) were aborted; the patient was G3 para 1 (P1) with full-term, normal delivery, and her intelligence was determined to be normal, as compared with children of the same age. Consanguineous relationships between the parents were found to exist for at least the previous three generations (Fig. 1A).

The findings of the physical examination were as follows: Blood pressure (BP), 90/60 mmHg; height, 120 cm; weight, 15 kg. No abnormalities were observed in the heart or lungs of the patient. The breast development stage of the patient was determined to be Tanner stage I (11). The mental status of the patient was evaluated as alert but weak. Hyperpigmentation of the skin and thin subcutaneous fat were noted (Fig. 1B). Normal muscle strength and tone were found in all extremities, but muscle volume was reduced and a flattening of the thenar eminence was observed. The abdominal reflex was positive, while the knee jerk, Achilles tendon and radial periosteal reflexes were found to be hyperreflexive. The patient was negative for meningism and positive for the Babinski sign on the right side. No abnormal gait was observed. The electroencephalograms (EEGs), brain MRI scan and evoked potential and nerve conduction studies showed no specific abnormalities. Esophagography showed achalasia of the lower esophagus at the cardia (Fig. 1C). Funduscopy indicated that the optic nerve discs were pale, suggesting optic nerve atrophy. The results of a bilateral Schirmer test were 0 mm, which led to a diagnosis of alacrima. Further medical history revealed that, not long after birth, the patient was unable to produce tears when crying. The suspicion that the patient was positive for AS was confirmed by genetic testing, which revealed a c.771delG mutation in exon 8 of the AAAS gene (6). The parents refused follow-up care for this patient, due to ‘familial reasons’.

#### Patient 2

A male patient, aged 2 years and 7 months, was admitted to the hospital on March 2, 2013 with a 1-year history of vomiting after either overeating or rapid eating. No apparent causes or obvious weight loss had been observed.

Six months prior to admission, the parents noted hyperpigmentation in the lips, photophobia and frequent blinking following birth. The patient was treated for dry-eye syndrome due to alacrima. There was no documented family history of adrenal gland disease. The parents did not have a consanguineous relationship and were in good health. The patient was G1P1 and the birth weight was recorded at 3.45 kg; the intellectual and physical development of the child was determined to be normal, as compared with children of the same age.

**Figure 2.**
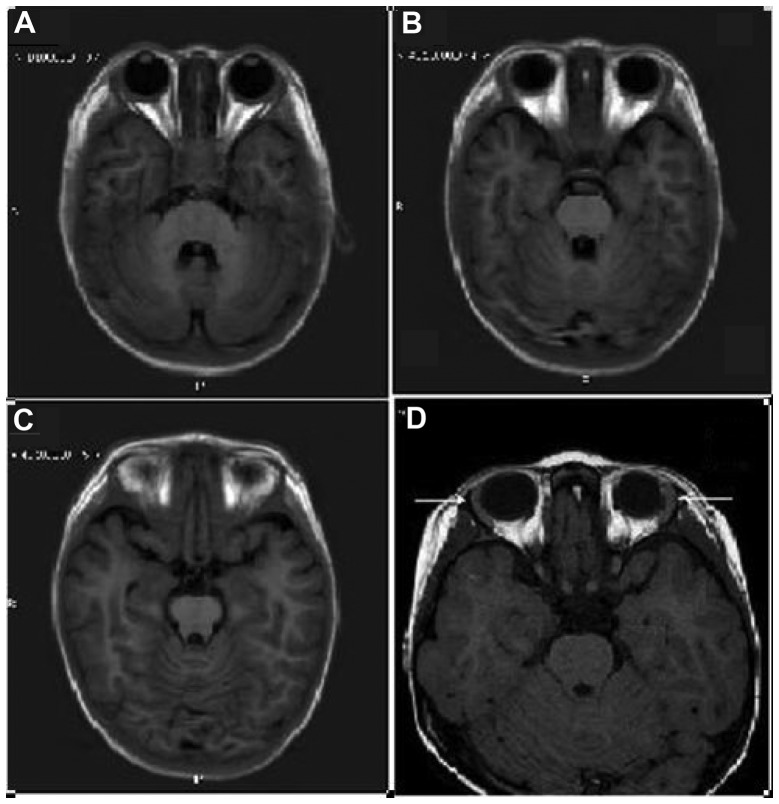
Lacrimal MR images of patient 2 compared with an image of a healthy child. (A-C) Axial T1-weighted, non-contrast MR images of patient 2, showing bilateral lacrimal gland agenesis. (D) Lacrimal gland MR image of a healthy child. An isointense internal signal is shown on the T1-weighted images (arrows indicate the lacrimal glands). MR, magnetic resonance.

**Figure 3.**
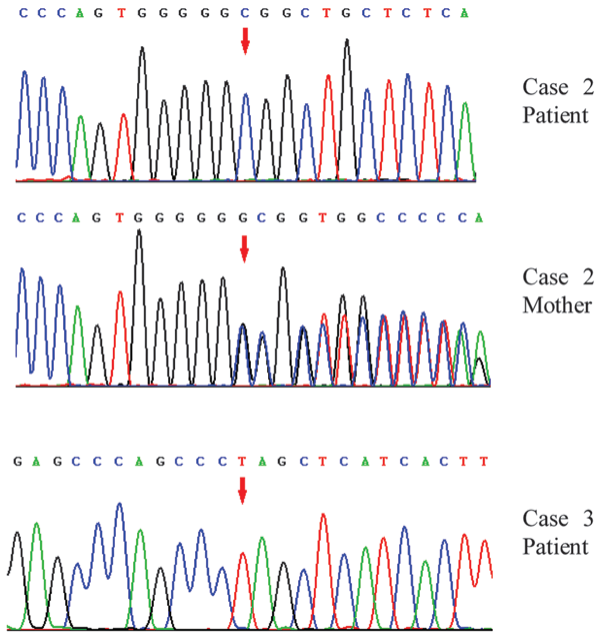
Gene sequencing data for cases 2 and 3.

The findings of the physical examination were as follows: BP, 86/50 mmHg; height, 92 cm; weight, 13 kg. The heart, lungs, abdomen, nervous system and external genitalia of the patient were found to be normal. Hyperpigmentation was observed over most of the body. Esophagography showed achalasia of the lower esophagus at the cardia. The patient was unable to produce tears when crying but refused to undergo the Schirmer test; therefore, an MRI scan (Fig. 2) was performed in order to confirm lacrimal gland hypoplasia and diagnose alacrima. The clinical diagnosis was AS. Genetic testing confirmed the presence of a c.771delG mutation in exon 8 of the AAAS gene. (Fig. 3). On day 3 after admission, the patient had a hypoglycemic episode and convulsion occurred due to an adrenal crisis. In response, the patient was treated with 100 mg/m^2^ hydrocortisone; the ACTH level decreased from >1,250 to 6.8 pg/ml (normal range, 0–46 pg/ml), and the hydrocortisone dosage was reduced to 20 mg/m^2^. Although the vomiting continued, without relationship with overeating, the patient was able to eat every day. The growth and nutritional status of the patient was found to be normal after 18 months of follow-up care.

#### Patient 3

A female patient, aged 4 years and 4 months, was admitted to the hospital with a 3-year history of vomiting small amounts following every meal. No obvious causes of the vomiting were identified. Approximately 2 years prior to admission, the patient exhibited hyperpigmentation of the lips, in addition to fatigue, with a slow/stagnating increase in weight and height.

One month prior to admission, the patient had a seizure and was diagnosed with secondary epilepsy, based on an abnormal EEG. The patient was successfully treated with Topamax but continued to vomit >10 times/day. The patient was G2P2 with full-term normal delivery and a birth weight of 3.35 kg. The intellectual and physical development of the patient was similar to that of healthy children of the same age. There was no family history of genetic diseases, and the parents were in a non-consanguineous marriage.

The findings of the physical examination were as follows: BP, 85/55 mmHg; height, 92.7 cm; weight, 12 kg. The patient was mentally alert, and no significant anomalies were observed in the heart, lungs, abdomen, nervous system or external genitalia. A generalized hyperpigmentation was observed. Esophagography showed achalasia of the lower esophagus at the cardia. In the hospital, the patient was observed not to produce tears when crying. The results of a bilateral Schirmer test were 0 mm, which led to the diagnosis of alacrima. Further medical history confirmed that the alacrima had manifested at birth. Following the diagnosis of AS, the patient was treated with hydrocortisone replacement therapy (25 mg/m^2^), and the vomiting was alleviated. Genetic testing confirmed a c.1366C>T mutation in exon 15 of the AAAS gene (Fig. 3). No additional follow-up information is available for this patient.

## Discussion

AS is a rare autosomal recessive congenital disease characterized by adrenal insufficiency (Addison's disease), failure of the lower esophageal sphincter to relax (achalasia) and an absence of tear secretion (alacrima). AS does not appear to be age-, ethnicity- or gender-specific (12), but varies widely in severity, with some patients developing no symptomology and others suffering a fatal outcome (13–15). Pediatric patients with AS often present with the classic triad of symptoms, while patients with the late-onset or adult-onset condition exhibit symptoms that involve the nervous system (16). The pathogenic gene for AS, AAAS, is located on chromosome 12q13 and consists of 16 exons, which encode the protein ALADIN (17). A variety of mutations scattered throughout the gene have been reported, with exon 3 being the exception, as no AS-related mutations have yet been reported therein. Of note, little correlation has been found among the genotype, phenotype and variable clinical expression of family members with AS (7,18).

In the present study, the early onset of the disease was a common characteristic among all 3 cases recorded. All patients had a history of excessive vomiting with no apparent etiology, which is a common symptom of AS in pediatric patients. Despite the fact that adrenal insufficiency was observed, all cases were misdiagnosed for >1 year. The patients were found to be unable to produce tears shortly after birth. Two of the patients (cases 1 and 3) were confirmed to have alacrima based on the results of the Schirmer test, while the other patient (case 2) refused to undergo the test. An MRI scan was instead performed to confirm lacrimal gland hypoplasia. Radiography of the upper digestive tract indicated delayed opening of the lower esophageal sphincter at the cardia, confirming the diagnosis of achalasia. Examination of patient 1 revealed tendon hyperreflexia and optic never atrophy, which indicated that this case of AS also involved a neurological manifestation, more commonly found in adult patients. Patients 2 and 3 did not exhibit any neurological symptoms. Vishnu *et al* (19) suggested that neurological symptoms may manifest in certain subgroups of patients with a less severe and chronic course of the disease. Based on the triad of adrenal insufficiency, achalasia and alacrima, all 3 patients described in the present study were diagnosed with AS.

Due to poor patient cooperation, patient 2 required an MRI scan. The lacrimal glands are located in the orbital lacrimal fossa, and their features on the T1- and T2-weighted MR images are similar to those of the extraocular muscles and cerebral gray matter density (20). Kim *et al* (20) described two cases of lacrimal gland agenesis in a single family. With regard to the present cases, the Schirmer test (bilateral) detected <1 mm of wetting in 5 min in patients 1 and 3, and the orbital MRI indicated the absence of both lacrimal glands in patient 2. Sahinoglu *et al* (21) performed lacrimal gland biopsy through a left superotemporal extraperiosteal approach. The histopathology showed no evidence of lacrimal gland tissue and an orbital MRI scan revealed the absence of both lacrimal glands. The fact that the study by Sahinoglu *et al* indicated a correlation between the MRI and biopsy findings provided the basis for the use of MRI to prove alacrima in the patient who would not cooperate with the Schirmer test in the present study.

AS is known to be caused by AAAS gene mutations that encode abnormal ALADIN proteins. ALADIN proteins belong to the tryptophan-aspartic acid-repeat-containing family of proteins (14) and contribute to the nuclear pore complex (NPC) (22). The ALADIN protein anchors to the cytoplasmic side of the NPC, and the mislocalization of a mutant ALADIN affects the exchange of nuclear material (23). Among the 16 exons of the AAAS gene, mutations have been identified in all except exon 3, which has a relatively shorter sequence (6,7). In addition, pathogenic mutations have been reported in introns 4, 5, 11 and 14 (24). Mutations include point, frame-shift and nonsense mutations, as well as DNA fragment deletions. Although no specific recognition of prominent hot-spot mutations has been found, there appears to be a relatively high frequency of mutations at the following loci: Exon 1 (c.43C>A, p.Q15K), exon 8 (c.787T>C, p.S263P) and intron 14 (IVS 14+1G>A) (6,7,24–27). The mutations in exons 1 and 8 have been reported in >20 families with no significant regional discrepancy (28–30). The same deletion in exon 8, a region that has been associated with a high frequency of mutations, was observed in patients 1 and 2. The most commonly observed mutation in exon 8 (c.787T>C, p.S263P) has been reported in cases predominantly from European countries (16); however, the mutation in exon 8 (c.771delG) found in the present study has only been reported in China, to date.

Among the mutations of the 3 cases reported in the present study, two of the patients exhibited the same homozygous point mutation and one exhibited a homozygous nonsense mutation. The patients in cases 1 (8) and 2 came from two unrelated families and exhibited the same homozygous point mutation, c.771delG, in exon 8, which caused a frame shift that subsequently generated a stop codon after 32 amino acids, resulting in a truncated protein, Arg258GlyfsX33, of 289 amino-acid residues. In both cases, the parents of the patients were found to carry heterozygous mutations. Case 3 had a homozygous c.1366C>T mutation in exon 15, which has also been reported in the United States in 2003 (31). This mutation causes a dysfunctional ALADIN protein truncated at amino acid 456. Residues 478–499 of the C-terminus of a normal ALADIN protein are essential for its membrane-anchoring function (14). Including the cases of the present study, 3 out of 4 AS cases reported in the Chinese Han population have exhibited the c.771delG mutation, suggesting that it may be a mutation specific to the Chinese (32).

AAAS, the pathogenic gene of AS, is widely expressed in human tissues, and the clinical manifestations of the disease are highly variable. There is currently no specific cure for the disease, only treatments that relieve its symptoms. The detection of AS through genetic analysis in pediatric patients that do not exhibit all the symptoms is likely to provide an opportunity for earlier diagnosis. Hormone replacement therapy to treat adrenal insufficiency appears to be the most effective treatment in the patients with AS. One case from the present study demonstrated cortisone resistance and required a higher dose of hydrocortisone therapy. Artificial tears can improve eye irritation, reduce eye blink rate and prevent eye infections and corneal ulcers in patients with alacrima. With regard to patients with achalasia, surgical intervention is generally not advocated, since there are less invasive and more effective treatment options, such as the application of a balloon to dilate the lower esophageal sphincter. If this treatment has no effect or fails, patients can be treated surgically with a lower esophageal ring myotomy. No effective treatments for the neuropathological symptoms of AS have been found. Due to the fact that autonomic dysfunction can cause the most severe symptoms, a neuropsychological and psychopathological assessment of patients with this syndrome is advised. Although genetic analysis of the AAAS gene in AS patients at a young age may facilitate an earlier disease diagnosis and comprehensive treatment, the problems in neurological and gastrointestinal systems require further investigation in order to facilitate the development of more effective novel treatments.

## Figures and Tables

**Table I.** Clinical summary for patients 1, 2 and 3.

Patient	Gender/age at admission (years)	Initial symptoms	Alacrima	Achalasia	Adrenal dysfunction	Neuropathy	Genetic mutation
1	F/7.25	Vomiting Hyperpigmentation	+	+	+	+	c.771delG Exon 8
2	M/2.60	Vomiting Hyperpigmentation	+	+	+	–	c.771delG Exon 8
3	F/4.30	Vomiting Hyperpigmentation	+	+	+	–	c.1366C>T Exon 15

## References

[1] Allgrove J, Clayden GS, Grant DB, Macaulay JC (1978). Familial glucocorticoid deficiency with achalasia of the cardia and deficient tear production. Lancet.

[2] Gazarian M, Cowell CT, Bonney M, Grigor WG (1995). The ‘4A’ syndrome: Adrenocortical insufficiency associated with achalasia, alacrima, autonomic and other neurological abnormalities. Eur J Pediatr.

[3] Sandrini F, Farmakidis C, Kirschner LS, Wu SM, TullioPelet A, Lyonnet S, Metzger DL, Bourdony CJ, Tiosano D, Chan WY (2001). Spectrum of mutations of the AAAS gene in Allgrove syndrome: Lack of mutations in six kindreds with isolated resistance to corticotropin. J Clin Endocrinol Metab.

[4] Bizzarri C, Benevento D, Terzi C, Huebner A, Cappa M (2013). Triple A (Allgrove) syndrome: An unusual association with syringomyelia. Ital J Pediatr.

[5] Mazzone L, Postorino V, De Peppo L, Vassena L, Fatta L, Armando M, Scirè G, Cappa M, Vicari S (2013). Longitudinal neuropsychological profile in a patient with triple A syndrome. Case Rep Pediatr.

[6] Papageorgiou L, Mimidis K, Katsani KR, Fakis G (2013). The genetic basis of triple A (Allgrove) syndrome in a Greek family. Gene.

[7] Huebner A, Kaindl AM, Knobeloch KP, Petzold H, Mann P, Koehler K (2004). The triple A syndrome is due to mutations in ALADIN, a novel member of the nuclear pore complex. Endocr Res.

[8] Gong CX, Wen YR, Zhao XL, Su C, Cao BY, Zhang X (2007). Allgrove syndrome in the mainland of China: Clinical report and mutation analysis. Zhonghua Er Ke Za Zhi.

[9] Sanger F, Nicklen S, Coulson AR (1977). DNA sequencing with chain-terminating inhibitors. Proc Natl Acad Sci USA.

[10] Dumić M, Barišić N, Rojnić-Putarek N, Kušec V, Stanimirović A, Koehler K, Huebner A (2011). Two siblings with triple A syndrome and novel mutation presenting as hereditary polyneuropathy. Eur J Pediatr.

[11] Marshall WA, Tanner JM (1969). Variations in pattern of pubertal changes in girls. Arch Dis Child.

[12] Nakamura K, Yoshida K, Yoshinaga T, Kodaira M, Shimojima Y, Takei Y, Morita H, Kayanuma K, Ikeda S (2010). Adult or late-onset triple A syndrome: Case report and literature review. J Neurol Sci.

[13] Ismail EA, TulliotPelet A, Mohsen AM, Al-Saleh Q (2006). Allgrove syndrome with features of familial dysautonomia: A novel mutation in the AAAS gene. Acta Paediatr.

[14] Cronshaw JM, Matunis MJ (2003). The nuclear pore complex protein ALADIN is mislocalized in triple A syndrome. Proc Natl Acad Sci USA.

[15] Toromanovic A, Tahirovic H, Milenkovic T, Koehler K, Kind B, Zdravkovic D, Hasanhodzic M, Huebner A (2009). Clinical and molecular genetic findings in a 6-year-old Bosnian boy with triple A syndrome. Eur J Pediatr.

[16] Dumic M, Barišic N, Kusec V, Stingl K, Skegro M, Stanimirovic A, Koehler K, Huebner A (2012). Long-term clinical follow-up and molecular genetic findings in eight patients with triple A syndrome. Eur J Pediatr.

[17] Weber A, Wienker TF, Jung M, Easton D, Dean HJ, Heinrichs C, Reis A, Clark AJ (1996). Linkage of the gene for the triple A syndrome to chromosome 12q13 near the type II keratin gene cluster. Hum Mol Genet.

[18] Luigetti M, Pizzuti A, Bartoletti S, Houlden H, Pirro C, Bottillo I, Madia F, Conte A, Tonali PA, Sabatelli M (2010). Triple A syndrome: A novel compound heterozygous mutation in the AAAS gene in an Italian patient without adrenal insufficiency. J Neurol Sci.

[19] Vishnu VY, Modi M, Prabhakar S, Bhansali A, Goyal MK (2014). ‘A’ motor neuron disease. J Neurol Sci.

[20] Kim SH, Hwang S, Kweon S, Kim TK, Oh J (2005). Two cases of lacrimal gland agenesis in the same family-clinicoradiologic findings and management. Can J Ophthalmol.

[21] Sahinoglu N, Tuncer S, Alparslan N, Peksayar G (2011). Isolated form of congenital bilateral lacrimal gland agenesis. Indian J Ophthalmol.

[22] Liu X, Mitchell JM, Wozniak RW, Blobel G, Fan J (2012). Structural evolution of the membrane-coating module of the nuclear pore complex. Proc Natl Acad Sci USA.

[23] Kind B, Koehler K, Lorenz M, Huebner A (2009). The nuclear pore complex protein ALADIN is anchored via NDC1 but not via POM121 and GP210 in the nuclear envelope. Biochem Biophys Res Commun.

[24] Babu K, Murthy KR, Babu N, Ramesh S (2007). Triple A syndrome with ophthalmic manifestations in two siblings. Indian J Ophthalmol.

[25] Palka C, Giuliani R, Brancati F, Mohn A, Di Muzio A, Calabrese O, Huebner A, De Grandis D, Chiarelli F, Ferlini A, Stuppia L (2010). Two Italian patients with novel AAAS gene mutation expand allelic and phenotypic spectrum of triple A (Allgrove) syndrome. Clin Genet.

[26] Brooks BP, Kleta R, Stuart C, Tuchman M, Jeong A, Stergiopoulos SG, Bei T, Bjornson B, Russell L, Chanoine JP (2005). Genotypic heterogeneity and clinical phenotype in triple A syndrome: A review of the NIH experience 2000–2005. Clin Genet.

[27] Brooks BP, Kleta R, Caruso RC, Stuart C, Ludlow J, Stratakis CA (2004). Triple-A syndrome with prominent ophthalmic features and a novel mutation in the AAAS gene: A case report. BMC Ophthalmol.

[28] Handschug K, Sperling S, Yoon SJ, Hennig S, Clark AJ, Huebner A (2001). Triple A syndrome is caused by mutations in AAAS, a new WD-repeat protein gene. Hum Mol Genet.

[29] Houlden H, Smith S, De Carvalho M, Blake J, Mathias C, Wood NW, Reilly MM (2002). Clinical and genetic characterization of families with triple A (Allgrove) syndrome. Brain.

[30] Prpic I, Huebner A, Persic M, Handschug K, Pavletic M (2003). Triple A syndrome: Genotype-phenotype assessment. Clin Genet.

[31] ReshmiSkarja S, Huebner A, Handschug K, Finegold DN, Clark AJ, Gollin SM (2003). Chromosomal fragility in patients with triple A syndrome. Am J Med Genet A.

[32] Lam YY, Lo IF, Shek CC, Tong TM, Ng DK, Tong TF, Choi MS, Lam ST, Ho CS (2006). Triple-A syndrome - The first Chinese patient with novel mutations in the AAAS gene. J Pediatr Endocrinol Metab.

